# Palliative Surgical Management of a Metastatic Lesion of the Tibia with Extension into the Popliteal Fossa Using Polytetrafluoroethylene Felt and Bone Cement

**DOI:** 10.1155/2020/8845173

**Published:** 2020-11-12

**Authors:** Kyriakos Papavasiliou, Konstantinos Asteriadis, Sousana Panagiotidou, Eleftherios Tsiridis

**Affiliations:** ^1^Academic Orthopaedic Department, Papageorgiou General Hospital, Aristotle University Medical School, Thessaloniki Ring Road, 56403 Nea Efkarpia, Greece; ^2^Center of Orthopaedics and Regenerative Medicine (C.O.RE.), Center of Interdisciplinary Research and Innovation (C.I.R.I.), Aristotle University Thessaloniki, Balkan Center, Buildings A & B, Thessaloniki, 10th km Thessaloniki-Thermi Rd, P.O. Box 8318, GR 57001, Greece

## Abstract

The treatment of metastatic bony lesions with the involvement of adjacent neurovascular structures presents a surgical challenge. We present—to the best of our knowledge—the first case of a patient suffering from a metastatic lytic lesion at the proximal tibia who underwent palliative treatment with the use of a polytetrafluoroethylene (PTFE) felt as a liner in order to preserve the adjacent vasculature and nerves. An 82-year-old female patient was diagnosed with multiple lytic bone metastases from renal cell carcinoma. One of these metastatic lesions was located at the proximal metaphysis of the left tibia. The lesion destructed the proximal metaphyseal part and the posterior cortex, and it was extending into the popliteal fossa. As a result, the patient was unable to bear weight. The patient was not fit to undergo radical operative treatment. As a means of palliative therapy, she underwent intralesional curettage and instillation of Poly-Methyl-Methacrylate (PMMA) bone cement using an alternative novel surgical technique with the use of a PTFE felt as a liner in order to protect the adjacent vasculature and nerves. This technique has proven to be successful in preventing cement leak into the popliteal cavity and efficient in allowing the patient to bear weight and walk independently until she demised 14 months later. The use of a PTFE felt as a liner, when treating lytic lesions, in order to protect the adjacent vasculature and nerves from PMMA leakage, is a helpful novel surgical option in cases when a radical treatment cannot be implemented.

## 1. Introduction

Patients presenting with secondary/metastatic bone sarcomas or carcinomas are a heterogeneous group and may be treated using the same regimens used for nonmetastatic sarcomas, provided that surgical resection of all sites of disease is deemed feasible [1, 2]. On the other hand, the treatment of metastatic bony lesions with the involvement of adjacent neurovascular structures presents a surgical challenge.

We present a case of a patient with a metastatic bony lytic lesion at the proximal tibia with extension into the popliteal cavity, who underwent palliative treatment with intralesional curettage and the subsequent instillation of Poly-Methyl-Methacrylate (PMMA) bone cement using an alternative novel surgical technique in order to protect the adjacent vasculature and nerves.

## 2. Case Presentation

An 82-year-old Caucasian woman was referred for evaluation and treatment to our department (a tertiary Orthopaedic and Sarcoma Referral Centre) by a consultant oncologist. Following staging, the patient had undergone open excision of her right kidney 10 months before her referral, due to renal cell carcinoma (RCC), followed by adjuvant chemotherapy. Approximately one month following her operation, the patient reported pain at her left proximal tibia. The gradually increasing severity of the pain had forced her to minimize her everyday-life activities. Being unable to bear weight, she was only capable of household walking with the use of a walker for a period of approximately 7 months before her referral. Eventually, she became bedridden.

**Figure 1:**
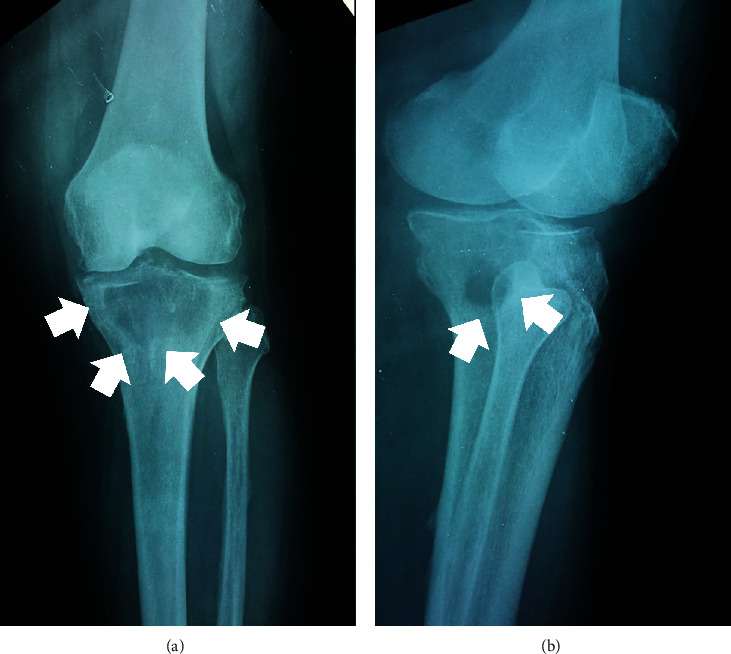
Preoperative radiograph ((a) anteroposterior, (b) lateral) of the left knee, depicting the existence of a large lytic lesion (white arrows) at the proximal tibia, extending into the popliteal cavity.

**Figure 2:**
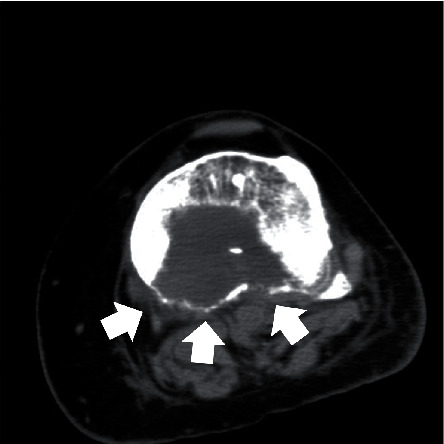
Preoperative axial CT-scan showing the destruction of the posterior cortex at the proximal part of the tibia (white arrows).

The patient's medical history was significant for coronary disease (had undergone cardiac by-pass 32 months before her referral) and impaired renal function, which severely deteriorated following her renal operation. Her physical examination revealed local tenderness at palpation at the proximal part of the right tibia. The patient underwent further examination with plain radiographs (Figure 1), a CT- and a MRI-scan (Figure 2), which showed the existence of a lytic lesion at the proximal tibia, extending into the popliteal cavity, due to the complete destruction of the posterior tibial cortex. Despite this extension of the lesion into the popliteal fossa, the lesion was abutting on the neurovascular structures and it did not encircle them. The diagnosis of a RCC metastasis was confirmed by CT-guided core needle biopsy. The patient subsequently underwent a bone-scan with Tc^99^ which showed evidence of multiple skeletal metastases (pelvis, lumbar and thoracic spine, and thoracic cage), even though she reported no pain at all other affected areas. Following a Multi-Disciplinary Meeting, the patient was offered two surgical options, either an above-knee amputation or a palliative limb salvage option. The latter involved extended curettage of the lesion, followed by instillation of PMMA bone cement to restore local anatomy, provide “bridging” between the subchondral part of the proximal tibia and the tibia metaphysis, alleviate pain, and reinstate weight bearing capacity.

## 3. Surgical Technique

**Figure 3:**
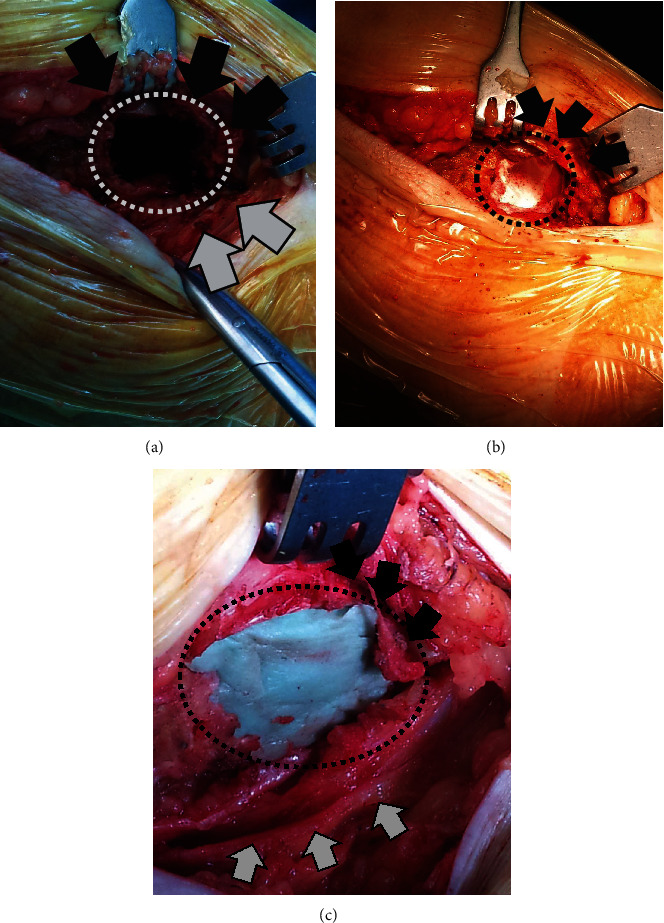
Intraoperative photos. (a) Through a typical posteromedial approach to the tibia, a cortical fenestration of approximately 3 × 3 cm was made to the proximal tibia (white dotted line). The lesion underwent extensive curettage under direct vision. (b) The polytetrafluoroethylene felt (black dotted line) was put in place in order to internally line the walls of the lytic cavity (black arrows point at the retracted pes anserinus). (c) Photograph taken immediately after the instillation of PMMA bone cement into the lytic cavity. PMMA bone cement filled the cavity and restored the medial tibial cortex continuity (black dotted line). Black arrows point at the retracted pes anserinus and grey arrows at the retracted gastrocnemius.

**Figure 4:**
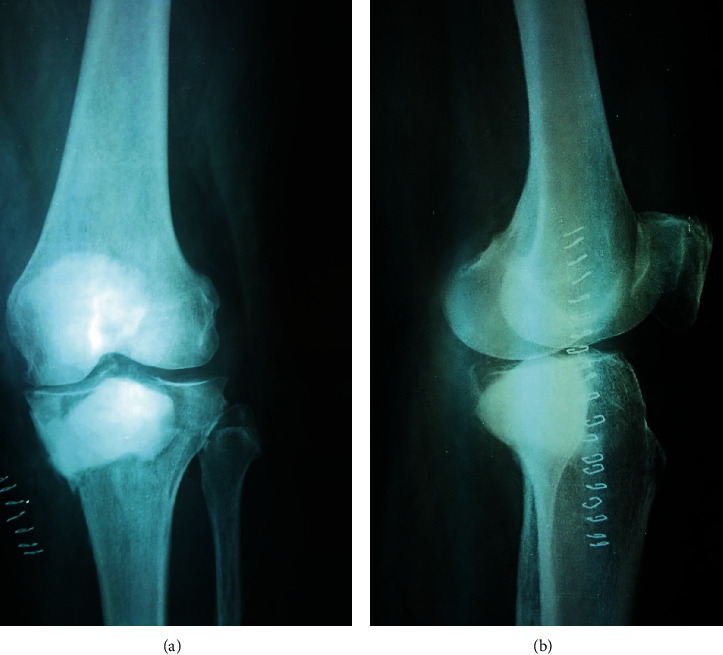
Immediate postoperative radiograph ((a) anteroposterior, (b) lateral) showing the complete containment of the PMMA bone cement within the lytic cavity.

The patient was operated on under epidural anesthesia in supine position and with the use of a tourniquet, following a simple elevation of the affected limb for a period of 10 minutes. A typical posteromedial approach to the proximal tibia was used. With the hip and knee in flexion, a slightly curved incision (starting from the joint line and extending to the posteromedial aspect of the tibia) was made. The fascia was opened and the pes anserinus exposed. The pes was retracted anteriorly, the gastrocnemius posteriorly and distally, and the medial edge of the tibia plateau was identified. Following confirmation of accurate access to the lesion with a C-arm, a cortical fenestration of approximately 3 × 3 cm was made to the proximal tibia, permitting direct access to the lytic cavity. Following meticulous curettage of the lesion, and in order to protect the PMMA bone cement from leaking to the adjacent popliteal cavity (with which the lesion was in direct contact), the cavity was lined with the use of a 15.2 × 15.2 cm polytetrafluoroethylene (PTFE) felt (BARD® Peripheral Vascular Inc., 1415 West Third Street Tempe, AZ 85281, USA). The lesional bone cavity was then completely filled with PMMA bone cement (Figure 3). Following meticulous hemostasis, the wound was sutured in layers, and a standard vacuum drain was used. Standard antibiotics prophylaxis was maintained for 2 days postoperatively. The drain was removed on the 2^nd^ postoperative day, and the patient has begun to bear weight to pain tolerance with the use of a walker soon after that (Figure 4). She was capable of full weight-bearing with the use of a cane six weeks later, the same period when her chemotherapy treatment resumed.

**Figure 5:**
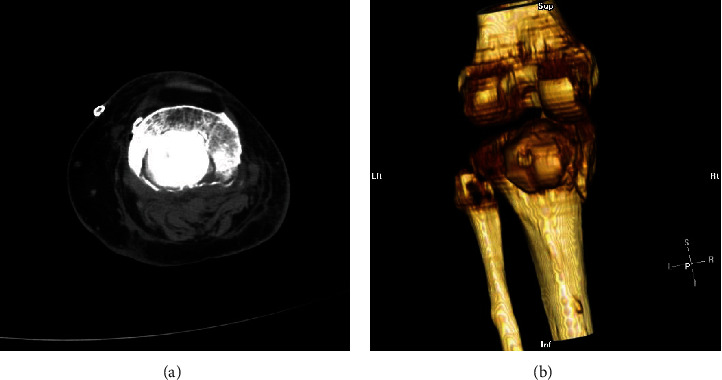
CT-scan ((a) axial, (b) 3-D reconstruction) at six months postoperatively, showing the containment of the PMMA bone cement within the lytic cavity, enabling the patient full weight-bearing.

**Figure 6:**
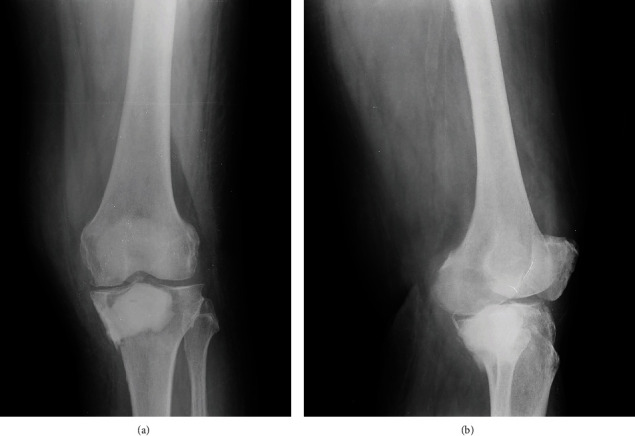
Plain radiograph ((a) anteroposterior, (b) lateral) at 12 months postoperatively showing containment of the PMMA bone cement and intact proximal tibia.

**Figure 7:**
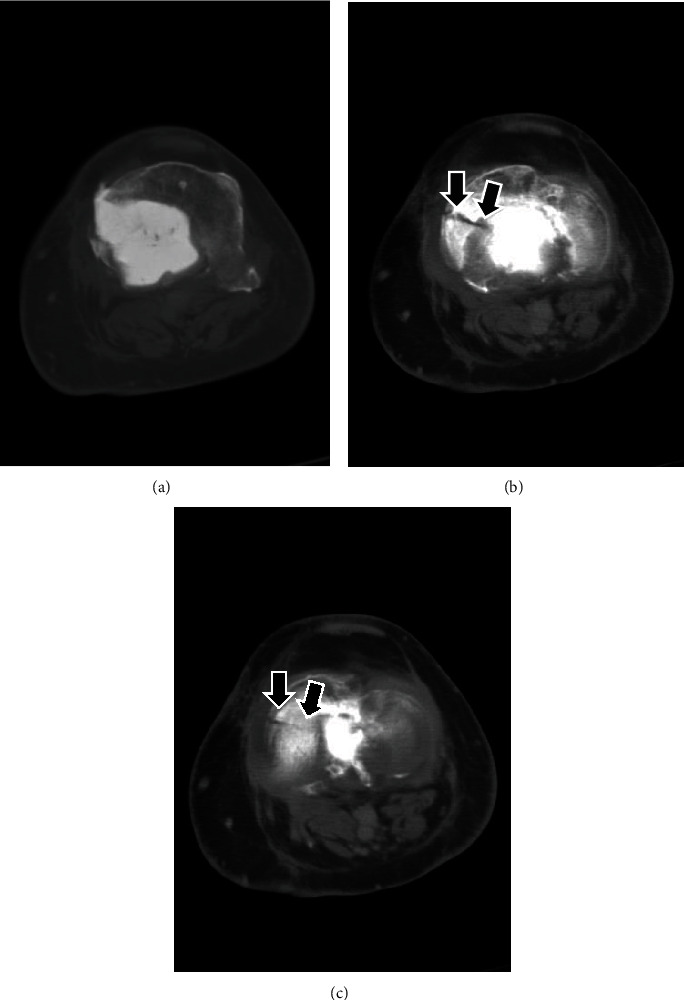
Axial CT-scan at 14 months postoperatively, moving from distal (a) to proximal (c). The PMMA bone cement is still contained; however, the proximal tibia of the patient sustained a pathological fracture (black arrows) after a fall.

**Figure 8:**
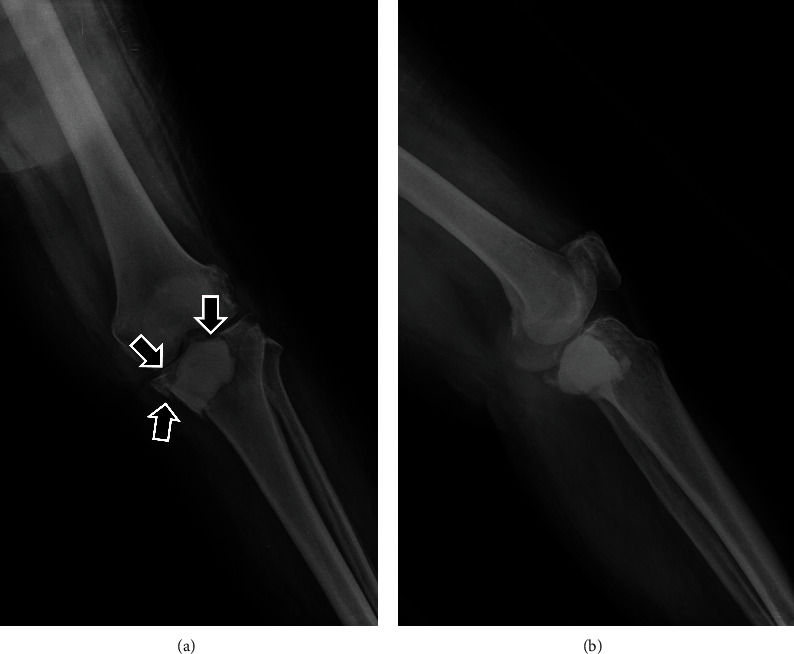
Plain radiograph ((a) anteroposterior, (b) lateral) at 14 months postoperatively showing the pathological proximal tibia fracture (black arrows). The PMMA bone cement is still contained.

**Figure 9:**
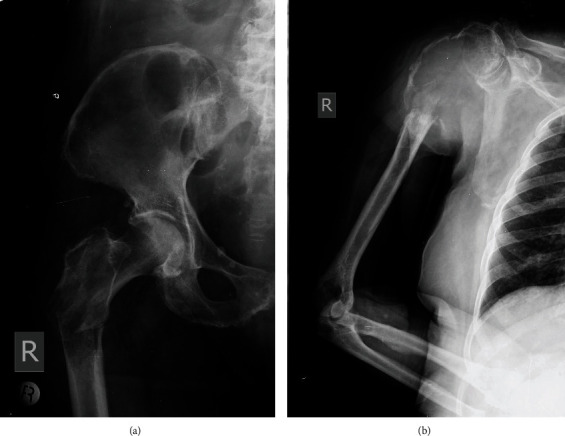
Plain radiographs ((a) anteroposterior of the right hip, (b) anteroposterior of the right shoulder and humerus) at 14 months postoperatively showing the pathological peritrochanteric and proximal humerus fractures the patient sustained following a fall.

The patient was followed-up at six (Figure 5) and 12 months postoperatively (Figure 6). During that period, she had developed multiple pulmonary metastases, partially responding to the chemotherapy regime administered. In both follow-up visits at the Orthopaedic Outpatient clinic, she was happy with the result of the operation, she was not bedridden, and she was still capable of full weight-bearing with the use of one cane, reporting no pain at her tibia or elsewhere at her skeleton. Unfortunately, at 14 months postoperatively, the patient fell and sustained a pathological fracture at the proximal left tibia, at an area adjacent to the operatively treated lytic lesion (Figures 7 and 8), together with a pathological peritrochanteric fracture at the contralateral hip and a pathological fracture at the right proximal humerus (Figure 9). Following her readmission at the orthopaedic department, the patient was asked whether she felt any pain at her shoulder and/or hip areas during the whole postoperative period. She admitted that she was occasionally feeling mild pain, with an onset of approximately 6 months following her operation, but she attributed it to her holding the cane with her right arm. The peritrochanteric fracture was treated with closed reduction and intramedullary nailing with a long Gamma-nail, and the humeral fracture was treated conservatively. Based on her decision, she underwent no further treatment for the proximal tibia fracture. The patient demised 2 months later at her home.

## 4. Discussion

Lytic bone lesions, being either the result of primary (benign or malignant) or metastatic tumors, may become a difficult diagnostic and therapeutic puzzle for orthopaedic surgeons, especially the ones who are not specialized in orthopaedic oncology. Several coexisting factors, such as the patient's age, medical history, and concomitant diseases, the location and the pathology of the tumor, the prognosis of the patient's primary disease (in metastatic tumors), and the existence of multiple metastases, may modify and influence the final decision regarding their treatment [3, 4].

Patients diagnosed with a solitary metastatic bone lesion, or with multiple resectable lesions, are considered as ideal candidates to be treated with radical excision of their lesion(s), in the context of a limb-salvage operation. This treatment modality seems to be the gold standard of treatment, provided that the primary disease has been controlled, the life expectancy of the patient is adequate, and that he/she is otherwise fit and well in order to undergo an operation of this magnitude [5, 6]. Palliative treatment, either conservative by radiotherapy alone or operative, is the remaining alternative option, in cases when one or more of these conditions are not met by the candidate patient [7, 8].

The use of PMMA bone cement is very often the treatment of choice in cases of lytic bone lesions [9], either as a palliative treatment method or as a final solution [10] (e.g., in benign bone cysts). The use of PMMA bone cement has several advantages, such as its instillation through a usually minimal approach, its fast application, the minimal blood-loss, the ability of the patient to bear weight immediately postoperatively in most cases, and the overall cost-effectiveness of the method, since it provides quality of life with minimal cost and morbidity.

Using PMMA bone-cement however in order to treat lytic lesions which extend to and/or are in direct communication with adjacent to the tumor vasculature and nerves may become a challenging task even for experienced orthopaedic surgeons. The high temperatures reached during the preparation of the PMMA bone cement, and its polymerization phase may bear catastrophic consequences if a direct contact between PMMA and the nerves and vessels cannot be avoided. As a result, in such cases, a certain “barrier” between the lytic cavity and the adjacent soft-tissues has to be put in place. A metallic cage or a metallic mesh often serves this purpose [11]. These materials however may be expensive, are often difficult to handle, require extensive approaches, and provide limited and often inadequate sealing of the cavity, compromising the final result.

The hereby reported patient was unable to undergo an extensive operative procedure due to her concomitant pathology. As a result, instead of using the abovedescribed and well-known methods, we decided to use a PTFE felt in order to seal the lytic cavity. PTFE felts are mainly used in cardiac surgery [12], most commonly as a patch, a buttress for sutures, and as a material to replace segments of the ventricular myocardium following resection. Furthermore, it is used for thoracic [13], vascular [14], aesthetic face surgery, and maxillofacial surgery [15]. Their main advantages are the facts that they are readily available, they come in different sizes, they can be easily cut by scissors and adjusted to the intraoperatively needed pattern, they are liquid-proof, they are heat-resistant, and—being extremely flexible—they can be easily inserted and adjusted to every cavity.

The use of a PTFE felt in order to seal the metastatic lytic cavity at the proximal tibia of our patient before the instillation of PMMA bone cement allowed us to perform a minimal invasive operation. Furthermore, it secured a complication-free postoperative result by preventing the leakage of the PMMA bone cement into the popliteal fossa, and it facilitated the complete filling of the lytic lesion by PMMA bone cement. Last but not least, this technique provided good quality of life to the patient, allowing full and painless weight-bearing with the use of one cane.

The use of a PTFE felt seems to be an efficient, easy to use, and cost-effective alternative method, which can be applied in cases when the sealing of a lytic bone lesion (either benign or malignant) is necessary before the instillation of PMMA bone cement.
